# Effects of Aluminum Oxide Addition on Electrical and Mechanical Properties of 3 mol% Yttria-Stabilized Tetragonal Zirconia Electrolyte for IT-SOFCs

**DOI:** 10.3390/ma15062101

**Published:** 2022-03-12

**Authors:** Justyna Chłędowska (Pleśniak), Jan Wyrwa, Mieczysław Rękas, Tomasz Brylewski

**Affiliations:** Faculty of Materials Science and Ceramics, AGH University of Science and Technology, Al. A. Mickiewicza 30, 30-059 Kraków, Poland; plesniak@agh.edu.pl (J.C.); jwyrwa@agh.edu.pl (J.W.); rekas@agh.edu.pl (M.R.)

**Keywords:** solid oxide fuel cells, tetragonal zirconia, ionic conductivity

## Abstract

Composite tetragonal zirconia (3Y-TZP) sinters with Al_2_O_3_ contents of 0, 1, 5, 10 and 15 mol% were obtained from a 3-YSZ powder prepared using the gelatin method, and the influence of alumina addition on the mechanical and electrical properties of the obtained sinters was investigated. Al_2_O_3_ was added via two different methods, namely during the preparation of the 3-YSZ powder and via impregnation using an alcohol solution of aluminum nitrate. The obtained green bodies were sintered for 2 h in air at 1773 K. The structure and morphology of the two series of sinters were investigated using XRD and SEM-EDS, their electrical properties were determined using impedance spectroscopy, and their hardness and critical stress intensity factor were measured using the Vickers indentation test. We established that both the amount of alumina and the method used to introduce it into the 3Y-TZP matrix significantly affect the physicochemical properties of the obtained polycrystalline material. The 3-YSZ/10 mol% Al_2_O_3_ sinter that had Al_2_O_3_ introduced during the preparation of the 3-YSZ powder was found to exhibit the most advantageous mechanical and electrical properties while still having sufficiently low porosity.

## 1. Introduction

A solid oxide fuel cell (SOFC) is an electrochemical device that generates electrical energy via the simultaneous oxidation of fuel and reduction of oxygen, both of which are supplied from an external source [1]. Unlike galvanic cells, namely batteries [2], solid oxide fuels do not need to have energy stored in them before generating current, and therefore their operating time is not reduced in the same manner. It is sufficient to supply the required fuel. In addition, SOFCs are characterized by high efficiency (45–60%) and a relatively long time of operation (up to 40,000 h). Last but not least, they are less expensive to manufacture than other types of fuel cells, such as PEMFC, since they do not require the use of noble metals, e.g., platinum.

A single cell consists of an anode, a cathode and a solid oxide electrode place between them and is responsible for the transport of oxygen ions [1,3,4]. Since the operating conditions of SOFCs are rather harsh and involve high temperatures and an oxidizing-reducing medium in the work space of both the cathode and the anode, the applied electrolyte materials must meet a number of strict technical and technological requirements. Most importantly, they need to exhibit high ionic conductivity (in excess of 10^−2^ S·cm^−1^) with no electronic conductivity component. They must furthermore have considerable thermochemical stability and low porosity and, consequently, gas-tightness and high mechanical strength [1,5,6].

A number of ceramic materials have already been successfully applied as efficient electrolyte materials, most notably LaGaO_3_ [1,7,8], δ-Bi_2_O_3_ [8], CeO_2_ [1,9] and ZrO_2_ [1,8,9]. Even though the first two of the previously mentioned oxides exhibit the highest ionic conductivity of the known oxide materials, they have not found widespread application thus far. This is because LaGaO_3_ readily reacts with the nickel found in the NiO-YSZ cermet, which is still commonly applied as the anode material in SOFCs; the product of this reaction is LaNiO_3_ [1,8]. 

On the other hand, bismuth oxide undergoes immediate reduction and loses its ionic conductor properties [8,10,11]. Electrolytes based on CeO_2_ also have major disadvantages—the electronic component of their conductivity becomes stronger as the oxygen partial pressure decreases, which is observed for the anode space of the cell, their performance deteriorates with time [1,8,12], and they are relatively expensive.

Materials that are still applied as solid electrolytes in SOFCs include cubic yttria-stabilized zirconia (C-YSZ) as well as tetragonal zirconia (3Y-TZP), although the application of the latter in this role is currently much less frequent—in modern planar anode-supported SOFC, it is applied far more often as a porous support material [4,13]. Fully stabilized zirconia (C-YSZ) exhibits high ionic conductivity in the temperature range of 1173–1473 K; however, it unfortunately has very low resistance to thermal shocks and low mechanical strength [14,15]. 

This is why recent years have seen research dedicated to the application of tetragonal zirconia stabilized with a 3 mol% addition of yttria as a solid oxide electrolyte in IT-SOFCs. This material is characterized by high mechanical strength (over 1 GPa) as well as high resistance to cracking (4–5 MPa∙m^1/2^) [16,17]. However, it exhibits lower total ionic conductivity compared to the cubic C-YSZ. When analyzing this in more detail, Badwal and Drennam [18] found that, at temperatures below 973 K, polycrystalline 3Y-TZP had higher ionic conductivity of the grain interior compared with C-YSZ, while the opposite was true with regard to the grain boundary conductivity, which was much lower for 3Y-TZP than for C-YSZ.

Various authors have attempted to elucidate this phenomenon, referred to as the “blocking effect”, and two main causes have been suggested [19,20,21,22]. The first of these is the presence of impurities—predominantly silicon dioxide, which segregates at grain boundaries, thereby, forming a thin insulating layer and inhibiting oxygen ion transport along grains [20]—whereas the other is the presence of a space charge layer near the grain boundaries, referred to as the dual Schottky barrier [22]. 

Although, from a structural perspective, the space charge region reaches into the grain interior, in terms of transport properties, it is part of the grain boundary. In such cases, the notion of an “electrical” grain boundary consisting of a lattice mismatch region and regions of space charge is usually applied. The thickness of this boundary is twice as large as the space charge region and is thus many times larger than the thickness of the structural boundary, exceeding 100 nm in some cases [23].

Butler and Drennan conducted microscopic observations of materials based on ZrO_2_-Y_2_O_3_-Al_2_O_3_ and they had shown that the alumina inclusions found in the 3Y-TZP matrix can effectively participate in a chemical reaction with silicon compounds that tend to accumulate at grain boundaries and purify them by forming mullite, which generally does not act as a barrier that would prevent the transport of oxygen ions in the respective regions of this polycrystalline electrolyte material [24]. On the other hand, Guo et al. [25] measured the electrical conductivity of 3Y-TZP via impedance spectroscopy, and they determined that aluminum ions may participate in the formation of complex defects involving oxygen vacancies and thus modify the Schottky barriers at grain boundaries. 

This effect is a strong indication of the positive influence of an Al_2_O_3_ on the electrical conductivity of grain boundaries in TZP. The above-cited works provided a starting point for a number of studies with the aim of establishing the effect of alumina on the electrical properties of zirconia, many of which have resulted in surprising or contradictory conclusions. According to some authors, Al_2_O_3_ improves the electrical conductivity of materials based on tetragonal zirconia [26], while other authors make the exact opposite claim [27]. 

Miyayama et al. [28] and X. Guo [29] reported a slight decrease in the conductivity of both grain interiors and grain boundaries when the alumina content in zirconia was below the solubility limit. On the other hand, the authors of [30,31] determined the grain boundary and grain interior electrical conductivity for unmodified 3Y-TZP as well as 3Y-TZP samples containing 0.25, 0.5 and 1.0 mol% of alumina and found the that the sample with a 0.5 mol% alumina content exhibited the highest values of these two parameters. Certain other studies found no changes in the ionic conductivity of grain interiors in zirconia with a small number of alumina inclusions or a slight decrease with a marked improvement in grain boundary conductivity. 

The latter leads to the so-called “scavenger effect” and Al_2_O_3_ inclusions displacing the impurities to grain boundary triple junctions [19]. If a large amount of alumina is added to the 3Y-TZP material and as a result the solubility limit is exceeded by a considerable margin, there is a pronounced decrease in the ionic conductivity of both grain interiors and grain boundaries [28,31,32]. The effective medium theory can be used to explain this drop [33]. According to the data presented in [28,33,34], the presence of aluminum cations in the 3Y-TZP crystal lattice is associated with an increase in the activation energy of both types of conduction; this increase is more pronounced for grain boundary conductivity.

The function played by the electrolyte material in an SOFC entails that it also needs to have certain mechanical properties in addition to electrical ones. The flexural strength of such materials needs to exceed 500 MPa, while their tensile cracking strength cannot be lower than 3MPa∙m^−2^ [35]. It is impossible to meet these criteria without modifying the zirconia material with inert corundum inclusions. The resulting material is a particulate composite; this composite varies in terms of the mechanical stress inside it depending on the area. The improved mechanical strength of composites consisting of the ZrO_2_-Y_2_O_3_ solid solution and Al_2_O_3_ inclusions stems from increased brittle fracture resistance and suppressed grain growth, and there are two phenomena that are associated with this—the removal of the glassy phase from phase boundaries and grain boundaries during the reaction with alumina and the subsequent modification of these boundaries, and another phenomenon that is related to additional mechanisms that absorb the elastic strain energy released when Al_2_O_3_ inclusions form. The most common mechanisms of this type in ceramic materials are crack bridging and crack path deviation [36].

The influence of alumina inclusions on the properties of tetragonal ZrO_2_-Y_2_O_3_ has been investigated by numerous authors [37,38,39,40]. The addition of alumina to yttria-stabilized tetragonal zirconia polycrystals allows ceramics with increased strength to be obtained [41]. According to Fukuhara [41], adding alumina increases the hardness of the material but is also associated with decreased fracture toughness (K_Ic_). Kihara et al. [42], in turn, achieved 17% and 15% increases in this parameter after adding 1 and 4 vol% of Al_2_O_3_ to 3Y-TZP, respectively. 

There is a direct correlation between the critical stress intensity factor, which is a measure of a material’s resistance to brittle fracture and the alumina content in the composites as well as their porosity. Since, as discussed earlier, adding alumina improves the mechanical properties of materials based on tetragonal zirconia, there was a rationale for a study of what addition is optimal in the context of application in electrolyte-supported intermediate-temperature SOFCs.

A review of the available literature shows that both the alumina content and the method used to synthesize the composite material affect its final properties in a very complex manner. The data that is currently available indicates that the nature of changes in transport and mechanical properties caused by the addition of alumina seem to depend on not only its concentration but also the way in which the tetragonal form of zirconia in the 3Y-TZP/Al_2_O_3_ system is generated and, consequently, how the microstructure of this system is modified. 

The most impactful parameters in this regard include the size of the inclusions, their location (grain boundaries or the interior of the grains of the matrix) and the grain size of the matrix. The method applied to synthesize aluminum-doped 3YSZ must therefore ensure that the obtained powders are highly homogeneous. In the case of the co-precipitation method, which is commonly applied to obtain 8YSZ or 3Y-TZP, this may be hard to achieve, since it does not guarantee the quantitative precipitation of aluminum. Potential alternatives include methods that remove the solvent from the solution, such as the gelatin method. Consequently, the objectives of the present study were to investigate the influence of both factors by preparing two series of composite materials based on yttria-stabilized tetragonal zirconia synthesized using the gelatin method. 

In the case of the first series, alumina was introduced into the 3Y-TZP matrix at the powder preparation stage. To provide additional data for comparison, a second series was also prepared; in this series alumina was introduced via impregnation with an alcohol solution of aluminum nitrate. For each series, several samples with different alumina content (0, 1, 5, 10 and 15 mol%) were prepared. The properties of the obtained materials were determined using structural, chemical, morphological, electrical and mechanical investigations.

## 2. Materials and Methods

### 2.1. Powder Preparation

The gelatin method was used to synthesize composite materials based on stabilized tetragonal zirconia polycrystals with a 3 mol% Y_2_O_3_ content (3Y-TZP) with alumina (Al_2_O_3_) inclusions, the composition of which can be expressed as (100 − x)3Y-TZP·xAl_2_O_3_ (x = 0, 1, 5, 10 or 15 mol%—equivalent to an alumina content of 0, 0.833, 4.194, 8.459 and 12.798 wt% respectively). For one series of samples, alumina was introduced into the 3Y-TZP matrix at the 3-YSZ powder synthesis stage. The samples from this series are referred to using the letter “G”.

The second series included samples for which the 3-YSZ was impregnated using the appropriate amount of an alcohol solution of aluminum nitrate. Letter “I” is used to refer to samples from this series. In addition, to differentiate the samples depending on their form, symbols P and S were added to the above-mentioned designations, with “P” representing powders and “S”—sinters. Finally, the number in the sample designation represents the alumina content in mole percent (Table 1).

The following reagents were used for sample synthesis, and they were all of analytical grade and supplied by Avantor Performance Materials Poland S.A. (Gliwice, Poland) or Sigma-Aldrich (Saint Louis, Missouri, USA): zirconyl nitrate (ZrO(NO_3_)_2_), yttrium(III) oxide (Y_2_O_3_), nitric(V) acid (HNO_3_), aluminum nitrate(V) nonahydrate (Al(NO_3_)_3_∙9H_2_O) and gelatin (C_102_H_151_O_39_N_31_). The individual preparation stages are described subsequently.

**Synthesis of 3-YSZ (GP-0):** The first stage of synthesis was the complete dissolution of 35 g of gelatin in 500 cm^3^ of distilled water, performed at a temperature of 333 K. Yttrium nitrate was prepared concurrently via the hot dissolution of yttria in concentrated nitric acid (HNO_3_). The appropriate amount of ZrO(NO_3_)_2_ solution with a concentration of 6.021 M and the Y(NO_3_)_3_ solution were then added to the prepared gelatin solution, and the resulting mixture was stirred using a SET GSM magnetic stirrer (Zamość, Poland) for 8 h. The obtained gel was dried at 373 K for 24 h and then calcinated for 1 h in air at 873 K. These particular conditions were selected based on the results of DTA/TG thermal analyses described in Supplementary Materials (Figure S1).

**Synthesis of G-series samples (GP-1 to GP-15):** The powders from this series were obtained in the same way as the GP-0 sample, however, with one major difference. An alcohol solution of aluminum nitrate(V) was added to the mixture containing the gelatin solution and the zirconium and yttrium ions in such a ratio as to obtain the intended nominal composition with an alumina content of 1, 5, 10 and 15 mol%. This mixture was then stirred for 8 h using a magnetic stirrer, and the obtained gel was treated as in the case of GP-0.

**Synthesis of I-series samples (IP-1 to IP-15):** The second procedure applied to obtain composite samples with the previously mentioned compositions involved the impregnation of the 3-YSZ (GP-0) powder obtained via calcination and milling with an ethanol solution of aluminum nitrate with the appropriate concentration. As part of this procedure, the 3-YSZ powder was placed in a glass container filled with an alcohol solution of aluminum nitrate(V), vigorously stirred for 24 h at 333 K using an ultrasonic stirrer and then dried for 24 h in an oven at 373 K. During the final step, the powders underwent 1 h of thermal treatment in the air at 873 K.

### 2.2. Sinter Preparation

To ensure that the sinters would be characterized by sufficient compactness, the calcinated powders were ground in an agate mortar and then formed into green bodies via uniaxial pressing at 50 MPa. The green bodies then underwent cold isostatic pressing at 250 MPa and were freely sintered. This process consisted of 2 h of sintering in laboratory air at 1773 K and yielded two series of sintered 3Y-TZP samples with an Al_2_O_3_ content of 1, 5, 10 or 15 mol% (GS-1 to GS-15 and IS-1 to IS-15) and a reference 3Y-TZP sinter without any alumina content (GS-0). The temperature at which the green bodies were sintered was selected based on the results of dilatometric measurements (Supplementary Materials, Figure S2 and Table S1).

### 2.3. Characterization of the Samples

The thermal decomposition of gel was conducted using the Netzsch STA 449 Jupiter F3 thermoanalyzer (Selb, Germany). This device made it possible to simultaneously record changes in the sample mass (TG) and the thermal effects associated with these changes (DTA). Each powder sample weighing 50 mg was placed in the platinum crucible. Measurements were conducted in dynamic conditions in synthetic air flowing at a rate of 10 dm^3^∙h^−1^ and with a heating rate of 2 K·min^−1^.

As the green bodies were being sintered, the kinetics of this process were examined using the Netzsch DIL 402C dilatometer (Selb, Germany). The samples were heated at a rate of 5 K∙min^−1^, using air at temperatures starting at room temperature and up to 1773 K.

The phase composition of the samples was evaluated by means of X-ray diffraction (XRD) performed using the PANatical X’Pert Pro PW 3710 diffractogram (Malvern, United Kingdom) and CuKα monochromatic radiation. The HighScore Plus application in conjunction with the software used to operate the X’Pert diffractometer and the ICDD database were used for phase identification. The lattice parameters were determined via Rietveld refinement, while the crystallite size was calculated using the Scherrer equation and the full width at half maximum (FWHM) of the (011) peak of tetragonal zirconia [43].

The morphology and chemical composition of selected regions of the samples were determined by means of an ultrahigh-resolution scanning electron microscope with a FEI field emission gun (Schottky emitter)—Nova NanoSEM 200 (FEI Europe, Eindhoven, the Netherlands)—equipped with an add-on for chemical composition analysis using energy-dispersive X-ray analysis (EDAX), manufactured by Genesis Spectrum, Mahwah, NJ, USA). 

To prepare the fracture cross-sections for morphological observations, the samples were polished and then thermally etched over 30 min. at 1623 K. The mean grain size and the grain shape coefficient in the investigated sinters were estimated based on SEM images processed using the Image J 1.52e application. The following dependence was used to calculate the previously-mentioned grain shape coefficient:

(1) $$O_{p}=4\pi \left(\frac{\mathrm{area}}{{\left(\mathrm{perimeter}\right)}^{2}}\right)$$

The theoretical range for this coefficient is from 0 (infinitely longpolygon) to 1 (perfect circle). The relative density of the examined samples was calculated by dividing the apparent density experimentally determined via the hydrostatic weighing of the samples in distilled water by their theoretical density. The total porosities were computed using the following equation:

(2) $$P_{c}=\left(1-\frac{d_{a}}{d_{\mathrm{XRD}}}\right)\;\cdot \;100\%$$

where P_c_—total porosity [%], d_a_—apparent density [g·cm^−3^] and d_XRD_—theoretical density [g·cm^−3^].

The theoretical densities for the sinters with alumina additions of 1, 5, 10 or 15 mol% were calculated using the rule of mixtures. For dual-phase 3Y-TZP/Al_2_O_3_ materials, i.e., when the amount of Al_2_O_3_ used was higher than what was required to remove all the silica according to Equation (3):

(3) $$3{\mathrm{Al}}_{2}O_{3}+2{\mathrm{SiO}}_{2}\Leftrightarrow {\mathrm{Al}}_{6}{\mathrm{Si}}_{2}O_{13}.$$

The theoretical density (d_XRD_) can be calculated from the following equation:

(4) $$d_{\mathrm{XRD}}=f_{3Y\text{-}\mathrm{TZP}}\cdot d_{3Y\text{-}\mathrm{TZP}}+\left(1-f_{3Y\text{-}\mathrm{TZP}}\right)\cdot d_{{\mathrm{Al}}_{2}O_{3}}$$

where f_3Y-TZP_—volume fraction of 3Y-TZP in the sample.

For a 3Y-TZP material that contains x mol% of unreacted Al_2_O_3_ (where x represents the initial mol% in the composition of a given 3Y-TZP/Al_2_O_3_ sample minus 0.25 mol%, which is the amount of alumina that had reacted with SiO_2_, as calculated from Equation (3)), f_3Y-TZP_ can be expressed using the following dependence:

(5) $$f_{3Y\text{-}\mathrm{TZP}}=\frac{\left(100-x\right)\cdot M_{3Y\text{-}\mathrm{TZP}}\cdot d_{{\mathrm{Al}}_{2}O_{3}}}{\left(100-x\right)\cdot M_{3Y\text{-}\mathrm{TZP}}\cdot d_{{\mathrm{Al}}_{2}O_{3}}+x\cdot M_{{\mathrm{Al}}_{2}O_{3}}\cdot d_{3Y\text{-}\mathrm{TZP}}}$$

where M_3Y-TZP_, d_3Y-TZP_, M_Al2O3_, d_Al2O3_—molar mass and theoretical density values for 3Y-TZP and Al_2_O_3_, respectively. The method used to determine the theoretical density of 3Y-TZP is described in the Supplementary Materials.

The Vickers hardness and fracture toughness of the samples were determined based on the results of measurements performed using the FV-700 hardness tester (Future-Tech Corp., Kanagawa, Japan). These involved the use of a diamond Vickers indenter in the shape of a pyramid with a square base and walls inclined at an angle of 136°. Loads of 9.81, 29.42 and 49.05 N were applied for 10 s. The critical stress intensity factor (K_IC_) was estimated by measuring the length of Palmqvist cracks originating at the corners of the indentations made during the Vickers hardness test using the formula by Niihara [44]:

(6) $$K_{\mathrm{IC}}=0.018{\mathrm{HV}}^{0.6}\cdot E^{0.4}\cdot a\cdot l^{0.5}$$

where a—one-half of the length of an indentation’s diagonal, l—crack length and E—Young modulus (a value of 200 GPa was assumed). The Vickers hardness values were calculated from the following formula:

(7) $$\mathrm{HV}=\frac{F}{S}\cdot 1.8544\cdot \frac{F}{d^{2}}\;\left[\mathrm{GPa}\right]$$

where F—applied force [N] and d = (d_1_ + d_2_)/2—mean length of the indentation’s diagonal [m].

The electrical conductivity of the samples was measured using electrochemical impedance spectroscopy (EIS) performed by means of computer-controlled Solartron FRA 1260 (Farnborough, Great Britain) frequency response analyzer with a 1294 dielectric interface. Samples in the shape of discs with a diameter of 10 mm and a thickness of ca. 1 mm were first coated with silver pasted and heated for 15 min at 923 K. The measurements were conducted in air at a temperature of 576 K and over the temperature range of 676–976 K, with a step of 50 K. Data were collected using the frequency range of 0.1 Hz–1 MHz. Impedance spectra were analyzed by means of the ZPLOT software supplied by Solartron with the equipment.

The following formulae were applied to calculate the grain interior (σ_b_) and total grain boundary (σ_total,gb_) electrical conductivity based on the determined resistance values and the dimensions of the samples:

(8) $$\sigma_{b}=\frac{L}{S\cdot R_{b}}$$

(9) $$\sigma_{\mathrm{total},\;\mathrm{gb}}=\frac{L}{S\cdot R_{\mathrm{gb}}}$$

where L—sample thickness [cm], S—cross-sectional surface area [cm^2^], R_b_—electrical resistance of the grain interior (b) [Ω] and R_total,gb_—total electrical resistance of grain boundaries (gb) [Ω].

Both bulk (σ_b_) and total grain boundary (σ_total,gb_) electrical conductivities as well as total (σ_total_) electrical conductivity understood as the sum of the former two types of conductivity can be treated as thermally activated ionic transport, which may be expressed as follows:

(10) $$\sigma =\frac{\sigma_{o}}{T}\exp\left(-\frac{E_{a}}{k_{B}\cdot T}\right)$$

where σ—grain interior/total grain boundary/total conductivity of the sample [S·cm^−1^], σ_o_—pre-exponential factor [(S·cm^−1^)·K], E_a_—activation energy of electrical conduction [eV], k—Boltzmann constant [eV·K^−1^] and T—absolute temperature [K].

When determining the electrical conductivity of grain interiors, their volume can be assumed to be equal to the sample volume and dependence (8) can be utilized, since any error this might lead to is negligible. On the other hand, to determine the “true” value of conductivity across gain boundaries, also referred to as specific grain boundary conductivity, the brick layer model should be applied [45]. 

In this model, the ionic conductor is composed of hexagonal grains separated by thin, flat grain boundaries, and it is assumed that, in this case, the grain size, expressed by the length of the cube’s side, is many times larger than the thickness of the boundary. It is furthermore assumed that current flow is unidimensional and that any curved surfaces near the cubes’ vertices can be disregarded.

The following dependence correlates the measured values of the electrical parameters of a material with the parameters that describe its microstructure [46]:

(11) $$C_{\mathrm{gb}}\cdot \frac{L}{S}=\varepsilon_{b}\cdot \varepsilon_{\mathrm{gb}}\cdot \frac{\delta }{d}$$

where C_gb_—grain boundary capacitance [C]; ε_b_, ε_gb_—dielectric permeability of grain interiors and grain boundaries, respectively [F·m^−1^]; δ—grain boundary thickness [m]; and d—average grain size [m].

Assuming that grain interior capacitance can be considered to be the same as the volume of the sample, the described model also entails that [47]:

(12) $$\frac{\delta }{d}=\frac{C_{b}}{C_{\mathrm{gb}}}\cdot \frac{\varepsilon_{\mathrm{gb}}}{\varepsilon_{b}}$$

where C_b_—grain interior capacitance [C].

Thus, the specific grain boundary conductivity, which is the average conductivity of the grain boundary, can be given as [48]:

(13) $$\sigma_{\mathrm{sp},\mathrm{gb}}=\frac{\delta }{d}\cdot \sigma_{\mathrm{total},\mathrm{gb}}$$

where σ_total,gb_—total grain boundary conductivity [S·cm^−1^], calculated based on sample geometry and grain boundary resistance values obtained from EIS measurements.

The grain boundary thickness (δ) was calculated from dependence (12), under the assumption that the dielectric properties of grains and the boundaries between them are approximately the same (ε_b_ ≈ ε_gb_). This assumption is justified when the chemical composition of both constituents of the microstructure is either the same or very similar. Sample GS-0 meets this criterion.

In the case of the grain boundary thickness of 3TZP-Al_2_O_3_ composites, in which aluminum accumulates at grain boundaries due to its low solubility in the crystal lattice of zirconia, calculations of the specific grain boundary conductivity should take into account the different dielectric properties of grains and grain boundaries. The dielectric constant of alumina assumed for grain boundaries is approximately 9 [49], whereas the dielectric constant of t-TZP grains is approximately 42 [50]. Consequently, the value of δ should basically be defined as the thickness of the intergrain layer containing alumina.

To apply dependence (12), it is necessary to know the values of the effective grain interior and grain boundary capacitances (C_b_ and C_gb_), usually determined from the following equation [48]:

(14) $$C_{i}={\left(R_{i}^{1-n_{i}}A_{i}\right)}^{\frac{1}{n_{i}}}$$

where R_i_—grain interior or grain boundary resistance, A_i_ and n_i_—parameters that characterize the impedance of the CPE_i_ elements, Z_CPE_:

(15) $$Z_{{\mathrm{CPE}}_{i}}=A_{i}^{-1}{\left(j\omega \right)}^{-n_{i}}$$

where i = 1 corresponds to the grain interior and i = 2 corresponds to the grain boundary.

## 3. Results and Discussions

### 3.1. Physicochemical Properties of the Powders

**Figure 1:**
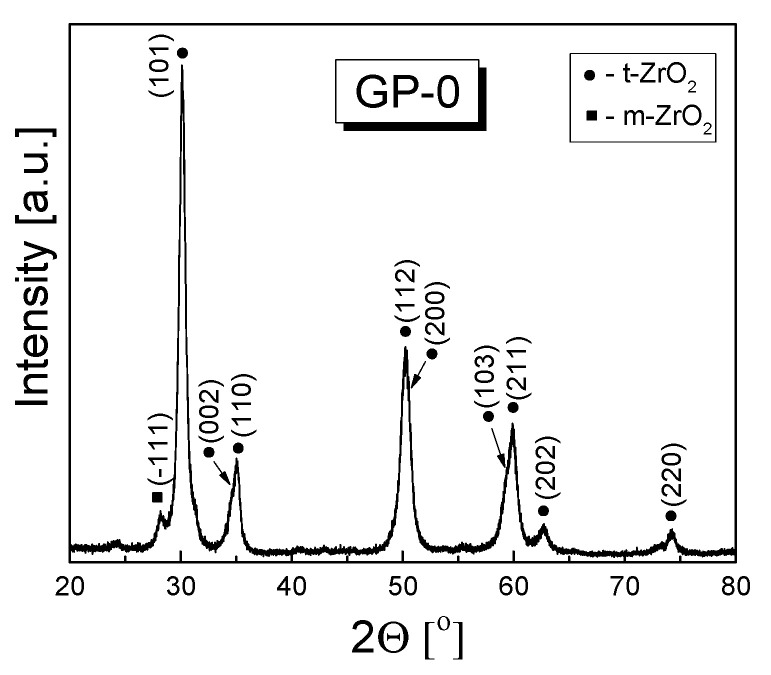
XRD pattern recorded for the 3-YSZ powder obtained by calcinating the precursor gel with no alumina addition for 2 h in air at 873 K (sample GP-0).

The Rietveld refinement of XRD patterns recorded for two series of powders (3-YSZ and 3-YSZ/Al_2_O_3_) calcinated for 2 h in air at 873 K revealed that they contained two polymorphs of zirconia, namely the cubic and monoclinic one. For reference, Figure 1 shows the diffraction pattern recorded for the GP-0 powder treated as mentioned above, while Table 2 lists the mass fractions determined for the detected zirconia polymorphs and the size of the tetragonal phase crystallites (D_(011)_).

It should be noted that no alumina precipitates were detected in the investigated powders. This may suggest that the alumina phase was present in the form of boehmite nanoparticles or it was amorphous due to the low temperature at which the gel precursors had been calcinated. According to the available literature data, boehmite decomposes to form alumina at ca. 773 K [51].

X-ray diffraction analysis also showed that the investigated powder were composed of nanocrystals, the size of which depends on the amount of added alumina and the addition technique. The largest crystallites, reaching ca. 13 nm, were observed for the powder obtained from the gel precursor without an alumina addition (GP-0), which in addition contained the largest mass fraction of the tetragonal phase of all studied samples.

As far as the remaining samples are concerned, the samples that had alumina introduced at the preparation stage (the GP series) had crystallites with a size ranging from 5.8 to 8.0 nm; the crystallites of the samples to which aluminum had been added via impregnation (the IP series) were larger (D_(011)_ = 8.6–11.6 nm). For the latter series, crystallite size decreased systematically as the amount of introduced aluminum increased. Overall, the IP powder samples were characterized by a small drop in the mass fraction of the tetragonal phase, while in the GP series, no such tendency was observed (Table 2).

**Figure 2:**
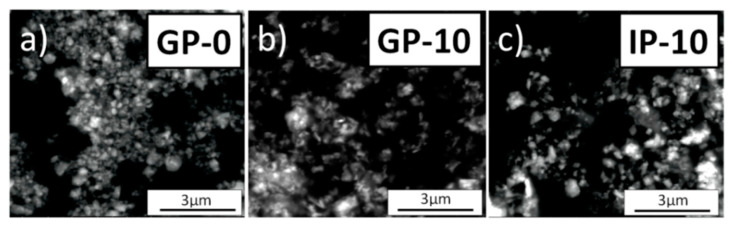
SEM micrographs of: (**a**) GP-0, (**b**) GP-10 and (**c**) IP-10 powders obtained by calcinating the precursor gel for 2 h in air at 1073 K.

Figure 2 shows the SEM images with the morphology of the GP-0, GP-10 and IP-10 powders obtained after 2 h of calcination in air at 873 K. The necessary analysis was conducted using a low vacuum detector (LVD).

The performed microscopic observations revealed that the investigated powders were composed of isometric nanocrystals with a tendency to agglomerate interspersed with single particles with an elongated, flat structure. The agglomerates were similar in shape regardless of the sample type. The mean agglomerate size for the GP-0 sample was 330 nm. On the other hand, the samples with a small addition of alumina—up to 5 mol%—had crystallite agglomerates with a size ranging from 50 to 250 nm. For higher alumina concentrations, mean agglomerate size increased, reaching a value of up to 310 nm.

### 3.2. Physical Characteristics of the Obtained Sinters

#### 3.2.1. Structure

**Figure 3:**
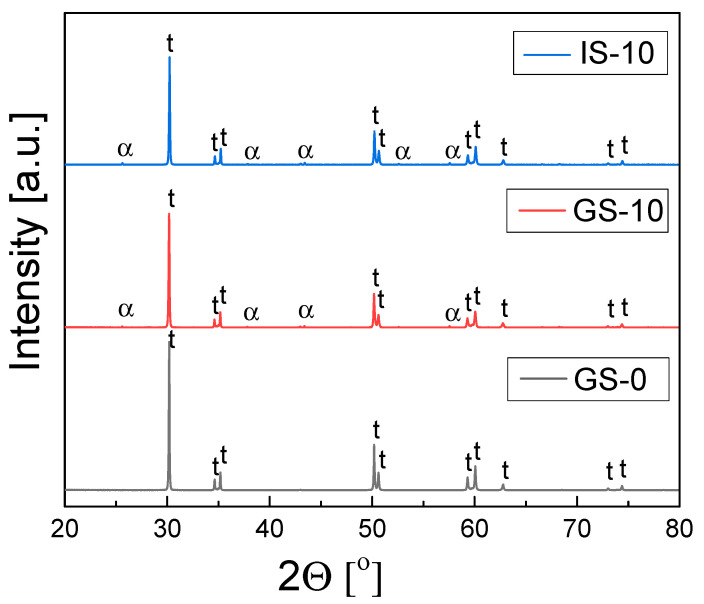
Diffraction patterns recorded for GS-0, GS-10 and IS-10 samples obtained via 2 h of sintering in air at 1773 K.

XRD phase composition studies of the sinters based on 3Y-TZP (GS-0) and the 3Y-TZP/Al_2_O_3_ composite samples from two series (GS-1 to GS-15 and IS-1 to IS-15) revealed that tetragonal zirconia was the predominant phase in the materials, with small amounts of monoclinic zirconia inclusions; the only exception was the IS-15 sinter, which contained a larger amount of the latter phase. In the case of composites from both series, α-Al_2_O_3_ was also found. All investigated sinters were obtained after 2 h of thermal treatment in air at 1773 K. Figure 3 shows the diffraction patters recorded for three sinters—the sinter without alumina in the 3Y-TZP matrix (GS-0) and the sinters with 10 mol% alumina content (GS-10 and IS-10).

A detailed analysis of the XRD data, which involved Rietveld refinement, showed that the mass fractions of alumina in the GS series of 3TZP/Al_2_O_3_ composites were approximately equal to those corresponding to their stoichiometric compositions. A similar situation was observed in the case of the IS-1 and IS-5 sinters, while for IS-10 and IS-15, the observed alumina content was higher than expected based on the stoichiometric composition. 

Two tetragonal variants with the P 42/nmc (137) space group were identified for all studied sinters and are referred to as t_1_and t_2_. These two phases differed slightly in terms of their lattice constants, and the mass fraction of t_1_ was significantly higher than that of t_2_. This is illustrated in Table 3, which lists the mass fractions of the identified phases, the lattice constants for the two tetragonal phases and the degree of tetragonality calculated from these constants for all investigated sinters.

It is worth noting that the largest amount of the monoclinic ZrO_2_ phase was observed for the sinter obtained from the powder that had 15 mol% of Al_2_O_3_ introduced via impregnation. The likely reason is an insufficiently high sintering temperature as indicated by the results of dilatometric analyses (Figure S2) and the comparatively low relative density of this sinter (Table 5).

In this case, the decreased sinterability of the material may be due to an overly high increase in the concentration of the stabilizer cation in zirconia’s crystal lattice in certain regions of the sample, which would affect the material’s chemical inhomogeneity. The remaining sinters contain negligible amounts of the m-ZrO_2_—at a level of ~0.5 wt% (Table 3). In this case, the disintegration of the monoclinic phase is followed by the sole growth of the tetragonal phase, and the associated process is related to the system reaching thermodynamic equilibrium and the increasing chemical homogeneity [52]. Another significant observations from the above-described research is the lack of significant differences in the values of lattice constants a and c determined for the two tetragonal phases in all investigated samples.

These differences are below the measurement error. Consequently, the chemical composition of these two tetragonal phases remains virtually constant regardless of the alumina content in the 3Y-TZP sinters, as demonstrated by the degree of tetragonality that represents the c/a ratio (Table 3 and Figure 5), which is likewise within the boundaries of experimental error. The only sample that does not follow this tendency exactly is the IS-15 sinter, as shown in Figure S3; the causes have already been described in this section.

In the event of the incorporation of a certain amount of alumina into the crystal lattice of tetragonal ZrO_2_—which may be expressed via the following defect reaction [53]:

(16) $${\mathrm{Al}}_{2}O_{3}\overset{{\mathrm{ZrO}}_{2}}{\to }2{\mathrm{Al}}_{\mathrm{Zr}}^{\prime }+V_{O}^{••}+3O_{O}^{x}$$

where as per the Krögera–Vink notation Al’_Zr_ represents the tripositive aluminum ion that substitutes the quadripositive cation of zirconium, $V_{O}^{••}$ is the fully ionized vacancy in the oxygen sublattice, while O_O_^x^ stands for an oxygen anion in a lattice position. The lattice constants of the unit cells of the tetragonal zirconia phases should be expected to decrease as a result of the substitution of Zr^4+^ in the octahedral configuration with Al^3+^, which has a smaller ion radius (98 pm vs. 67.5 pm, respectively) [54]. The fact that this effect was not observed leads to the conclusion that nearly all of the added Al_2_O_3_ segregates at the grain boundary of 3Y-TZP.

#### 3.2.2. Morphology and Chemical Composition

To establish how the performed alumina addition affects the microstructure of sintered tetragonal zirconia in the 3Y-TZP/Al_2_O_3_ composite obtained from the 3-YSZ powder synthesized via the gelatin method, the thermally etched polished cross-sections of the corresponding samples were examined using SEM-EDS. 

**Figure 4:**
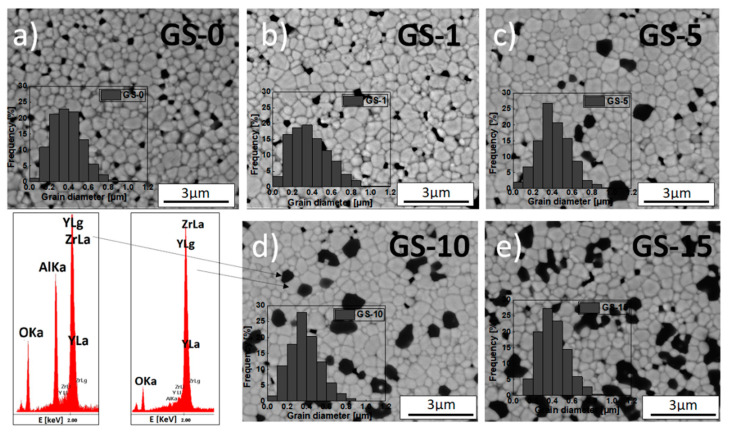
SEM micrographs of fractured sections of GS samples: (**a**) GS-0, (**b**) GS-1, (**c**) GS-5, (**d**) GS-10 and (**e**) GS-15 and the results of EDS point analyses for the area corresponding to the 3Y-TZP matrix and alumina inclusions in the GS-10 sample. Alumina addition performed via the gelatin method. All sinters obtained after 2 h of sintering in air at 1773 K. Inset: grain size distribution.

**Figure 5:**
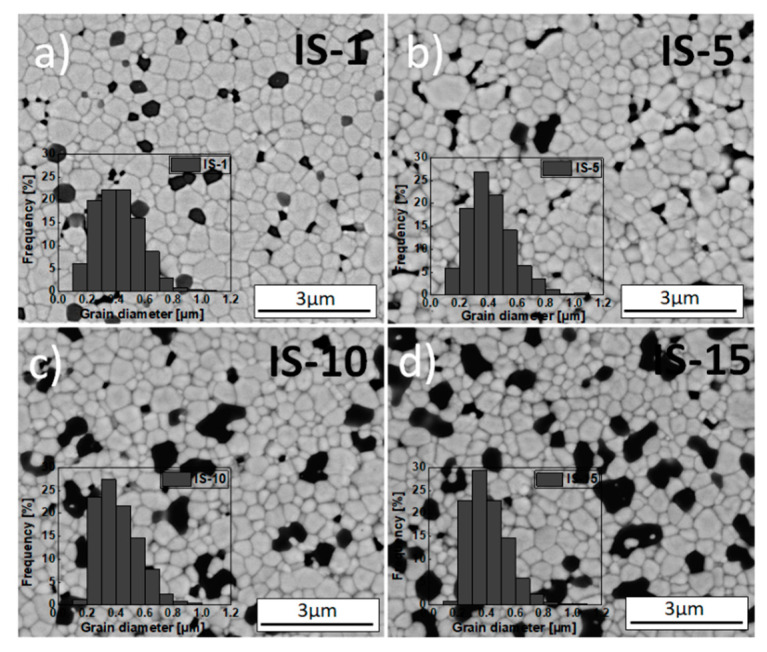
SEM micrographs of fractured sections of IS samples: (**a**) IS-1, (**b**) IS-5, (**c**) IS-10 and (**d**) IS-15. Alumina addition performed via impregnation. All sinters obtained after 2 h of sintering in air at 1773 K. Inset: grain size distribution.

The microstructural parameters that were determined directly from these observations included the surface and grain circumference, and these parameters were used to compute the diameter of the flat grains of both the matrix and inclusions and their circularity. Figure 4 and Figure 5 include SEM micrographs that show the microstructure of the 3Y-TZP (GS-0) sample and the series of samples obtained using different methods—3Y-TZP/Al_2_O_3_ (GS-1 to GS-15 and IS-1 to IS-15)—after 2 h of sintering in air at 1773 K.

Figure 4 and Figure 5 also show the grain size distribution in the matrix (inset). The presented micrographs were taken using a back-scattered electron detector (BSED). EDS chemical point analyses were also conducted for selected regions of samples—the result for the GS-10 sample is included in Figure 4.

Table 4 lists the values of mean size of grains in the 3Y-TZP matrix and the alumina inclusions and their circularity for all studied samples. The above-described microscopic observations revealed that all obtained sinters consist of uniformly distributed grains of the 3Y-TZP matrix (indicated by the brighter structures in the micrographs) that varied in size and were approximately isometric (Figure 4 and Figure 5, Table 4).

The size distribution of matrix grains was unimodal and relatively narrow. It was also characterized by the typical asymmetry towards larger grains. In some regions of the investigated sinters, small pores can be seen. The formation of these pores was facilitated by the presence of hard agglomerates during the sintering of green bodies.

In the sintered composites containing alumina, the vast majority of alumina inclusions (dark patches in the SEM micrographs) were located at the grain boundaries. Small amounts of inclusions, generally of very small size, were also found in the interior of zirconia grains, which was caused by the shift of the migrating grain boundary due to an anchored inclusion [55].

It is also worth noting that, for the composites containing no more than 10 mol% of Al_2_O_3_ (GS-1 to GS-10 and IS-1 to IS-10) the previously mentioned inclusions of alumina were distributed with relative uniformity across the entire volume of the matrix (Figure 4b–d and Figure 5a–c). In the case of the sinters with higher concentration of alumina, namely GS-15 and IS-15, areas in which these inclusions had accumulated can be seen (Figure 4e and Figure 5d).

As indicated by the data shown in Table 4 and also included in Figure S4 for the sake of clarity, the mean grain size in the 3Y-TZP matrix in samples from the IS series decreases systematically as Al_2_O_3_ concentration increases and, moreover, slightly exceeds the corresponding values for the GS series samples, for which this tendency is generally not observed. In the case of alumina inclusions, both series of samples saw a comparable, systematic increase in mean size with alumina concentration. In addition, the inclusions formed for samples with high alumina content (≥10 mol%) were significantly larger than the corresponding grains of the matrix (Table 4, Figure S4b). 

It is worth noting that increased alumina concentrations were associated with slightly more narrow grain size distributions and, moreover, the maxima shifted towards smaller grains; this was true for both series of composites. This might indicate that the alumina inclusions restricted the mobility of grain boundaries and the growth of the grains of the matrix [56,57]. However, this effect was far more pronounced for the IS series of samples than for the GS one (Table 4, Figure S4a); the results for the IS series were consistent with the data reported by Nevarez-Rascon et al. [58].

The circularity values calculated for the grains of the matrix in the GS series are slightly lower than those for IS samples, and furthermore they did not depend on the alumina content. As far as the alumina inclusions are concerned, their mean circularity was lower than for the grains of the matrix. In this case, Al_2_O_3_ was again found not to affect grain shape (Table 4). The determined circularity, on the other hand, had a significant effect on the migration of grain boundaries in the investigated materials. Pawłowski and Bućko [59] demonstrated that the migration force of grain boundaries was highly dependent on their curvature, which was in turn related to the circularity. 

Taking into account the above the structure of grain boundaries in zirconia-based materials should be considered to have a dynamic state. It is widely known that coarse-crystalline Y_2_O_3_-ZrO_2_ solid solutions with a tetragonal structure have a major flaw, namely the tetragonal-to-monoclinic phase transition that occurs in the temperature range of 473–573 K and is accompanied by considerable changes in molar volumes [52]. This effect causes the mechanical properties of the material to grow worse and reduces electrical conductivity. This phenomenon was observed when grain size in the 3Y-TZP material exceeded the critical value of ca. 0.3 μm [60]. Consequently, the sinters from the GS series should be considered to be the most stable ones—GS-10 in particular.

#### 3.2.3. Density and Porosity

The density of the sinters, which is a measure of the sinterability of powders, was determined based on their relative density and total porosity. Table 5 lists the measured values of apparent, XRD and relative density as well as the total porosity for the unmodified 3Y-TZP sinter (GS-0) and the sintered 3Y-TZP/Al_2_O_3_ composite samples from the GS and IS series. 

To determine the relative density—expressed as the ratio of apparent density to theoretical density—it is necessary to know the value of the apparent density determined experimentally from the geometric dimensions and the mass of each sinter and the theoretical densities for sinters with alumina additions of 1, 5, 10 or 15 mol%, which were calculated based on the rule of mixtures (discussed in detail in Section 2.3). Equation (2) was used to calculate the total porosity.

The data presented in Table 5 shows that the relative density of the sinters varied and was to a large degree dependent on the concentration of alumina as well as the method used to add it to the 3Y-TZP matrix. The relative density values were generally higher for the GS sinters than for the IS ones—with the exception of the GS-1 sinter, which had the lowest relative density. The morphological observations of the sinters described in Section 3.2.2 confirm this. The lack of direct correlation between these data and the alumina content in either series of 3Y-TZP/Al_2_O_3_ is worth emphasizing. The highest relative density of 99% and thereby the lowest porosity was observed for the 3Y-TZP sinter obtained by adding 10 mol% of Al_2_O_3_ via the gelatin method.

Assuming that at a relative density of above 96% the entire porosity is closed, it can be concluded that the GS-10 sinter should be gas-tight and therefore suitable as an electrolyte material for application in intermediate-temperature solid oxide fuel cells (IT-SOFCs). The remaining sinters can act as base materials for the synthesis of oxide materials with mixed ionic–electronic conductivity.

#### 3.2.4. Mechanical Properties

The critical stress intensity factor (K_IC_), which is an appropriate measure of fracture toughness for ceramic materials, was determined based on hardness tests conducted with the used of the Vickers indenter. A applied load of 9.81 N did not cause fractures, while loads of 29.42 N and 49.05 N resulted in Palmqvist cracks; the cracks were used to calculate the K_IC_ values from Equation (6) proposed by Niihara et al. [55].

Table 6 shows the Vickers hardness (HV) values for the three tested loads—9.81, 29.42 and 49.05 N—and the K_IC_ values calculated for the latter two loads for all samples from the GS series and all samples from the IS series with the exception of sample IS-15. In the case of IS-15, the indenter sank into the sample upon contact, which made it impossible to measure its hardness.

As follows from the data listed in this table, the HV values of the investigated sinters depended not only on the alumina concentration but also on the addition method. With the exception of GS-1, sinters from the GS series had a higher hardness than those from the IS series. The 1 mol% alumina addition used to obtain the GS-1 sinter reduced its hardness compared to the GS-0 one, as might have been expected based on its relative density. Of the IS series, IS-1 had the lowest hardness. For both series of composite samples, higher alumina concentrations resulted in increased amounts of inclusions and, subsequently, higher hardness. Hardness peaked for 10 mol% of alumina and no further increase was observed for the GS-15 sinter.

As far as the critical stress intensity factors are concerned, they were higher for the IS series of sinters than both the factor determined for GS-0 and the factors determined for the GS composite samples. No significant correlation between the concentration of alumina inclusions in the 3Y-TZP matrix and the KIC value was found for either series of composites. 

Of the sinters in the respective series, GS-1 and IS-1 sinters had the highest brittle fracture toughness at the load of 49.05 N; the corresponding values of this parameter were 21% and 39% higher than the brittle fracture toughness of the GS-0 sinter. Similar values ranging from 6 to 12 MPa·m^1/2^ were reported in the literature for Y-TZP and Ce-TZP materials [61]. This proves that polycrystalline tetragonal zirconia materials can be applied at both low and intermediate temperatures.

The alumina inclusions in the 3Y-TZP material’s matrix are generally thought to increase brittle fracture toughness via crack deflection caused by the residual stress field. The stresses in question can be attributed to differences in the Young moduli of the two phases and their thermal expansion coefficients (TECs). 

3Y-TZP has a higher TEC than the Al_2_O_3_ inclusions; whereas, ductile stress is generated in the matrix, compressive stress occurs in the inclusions. Upon the propagation of a crack throughout composite materials, such as 3Y-TZP/Al_2_O_3_, the stress field in the matrix “attracts” this crack’s vertex to the inclusion. The likely results is that cracks along grain boundaries are more prevalent than cracks across the grains of the matrix [62].

#### 3.2.5. Electrical Properties

**Figure 6:**
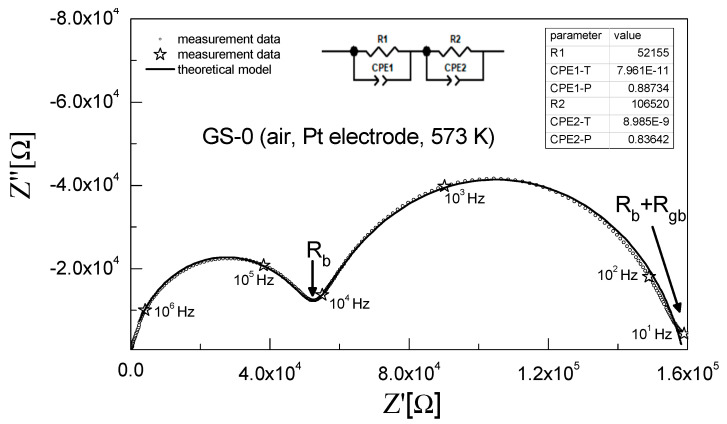
Impedance spectrum recorded at 573 K for the 3Y-TZP sample with no alumina addition (GS-0) and the curve fitted to experimental data.

The ionic conductivity of the obtained sinters was investigated using electrochemical impedance spectroscopy (EIS). All relevant details are described in Section 2.3. Figure 6 shows a Nyquist impedance plot recorded at 573 K for the unmodified 3Y-TZP sinter (GS-0) and the curve fitted based on the assumed equivalent circuit.

The spectrum in this impedance plot had the shape of two partially overlapping semicircles. As per the generally accepted interpretation, the phenomena taking place in the grain interiors, grain boundaries and electrodes are observed for different frequency ranges. The impedance spectrum for the range of high frequencies (first semicircle) is associated with the electrical conductivity of the grain interior, whereas for lower frequencies (second semicircle) a response originating from the electrical conductivity of grain boundaries is observed [49,63].

The inset in Figure 6 shows the equivalent circuit assumed for the analysis of the measured data. It consists of two resistors connected in series and representing the electrical resistance of grain interiors (R_1_) and grain boundaries (R_2_). In addition, parallel constant phase elements (CPE_1_ and CPE_2_) are connected in parallel to certain elements of the circuit. The applied equivalent circuit was determined based on the measurement data for samples exposed to 673 K. 

At higher temperatures, an electrical response associated with electrode polarization was also observed. Consequently, the equivalent circuit used for the analysis of impedance spectra at such temperatures had three resistors connected in series, with each of these resistors connected in parallel to a CPE; the CPE for the lowest frequency corresponded to electrode polarization [49].

The obtained electrical resistance values and the geometric dimensions of the samples were used to determine the grain interior (σ_b_) and total grain boundary (σ_total,gb_) electrical conductivities as well as the total (σ_total_) electrical conductivity of all samples for the temperature range of 573–973 K. Equations (8) and (9) were used for this purpose.

**Figure 7:**
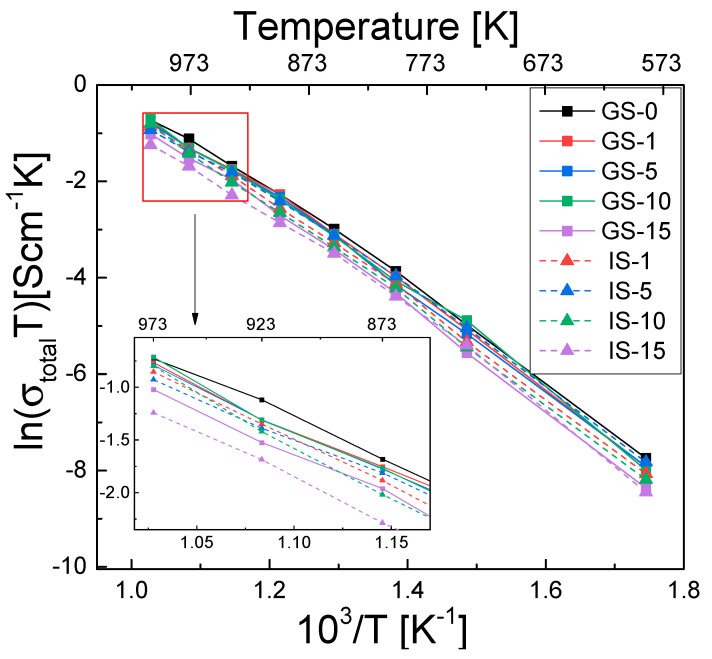
Temperature dependence of total electrical conductivity in the form of Arrhenius plots for all investigated sinter samples. Inset: Arrhenius plot in the temperature range of 873–973 K.

Figure 7 shows the temperature dependence of total electrical conductivity (σ_total_) for all samples, as measured for the previously mentioned temperature range.

We found that the value of this parameter increases together with temperature, suggesting that electrical conductivity in the samples has a thermally activated character. The GS-0 sinter exhibited the highest electrical conductivity across the entire measurement range; this was due to the absence of the monoclinic phase (Table 3)—its electrical conductivity is two orders of magnitude lower than that of the tetragonal phase [60]. 

The composite sinters with alumina content of up to 10 mol% (GS-1 to GS-10 and IS-1 to IS-10) had only slightly lower electrical conductivity. The samples with the highest alumina concentration (GS-15 and IS-15) exhibited the lowest electrical conductivity. The presence of the monoclinic phase is the likely reason for this tendency. The alumina addition was also found to affect electrical conductivity. For alumina additions of 1–10 mol%, the GS sinters were characterized by higher conductivity, while the opposite was true for the samples obtained by adding 15 mol%.

**Figure 8:**
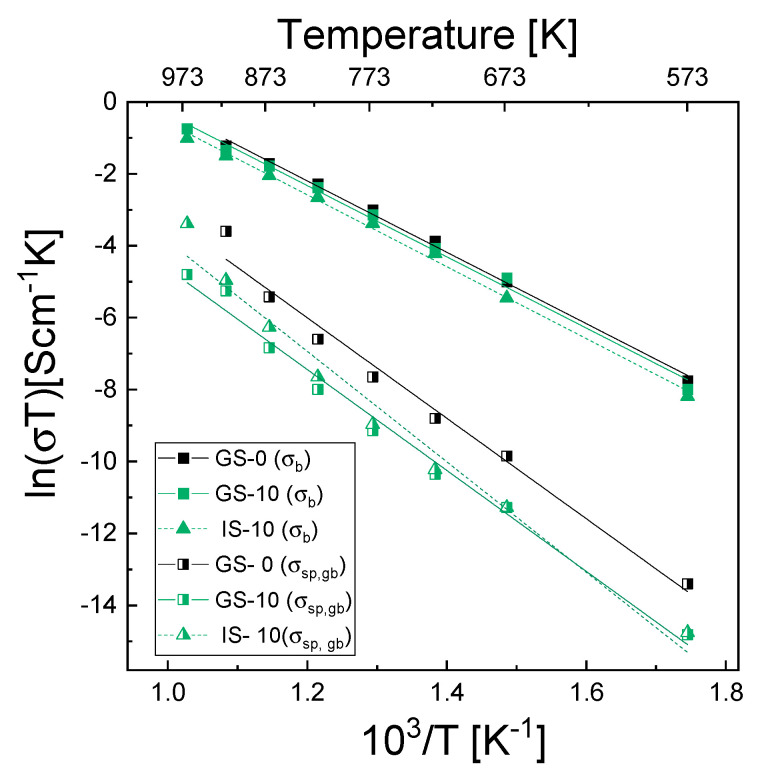
Temperature dependences of the electrical conductivity of grain interiors (σ_b_) and of the specific electrical conductivity of grain boundaries (σ_sp,gb_), expressed in the form of Arrhenius plots for samples GS-0, GS-10 and IS-10.

Figure 8 shows the temperature dependences of the electrical conductivity of grain interiors (σ_b_) and the specific electrical conductivity of grain boundaries (σ_sp,gb_); although both of these dependences were determined for all samples, for the sake of clarity only plots for samples GS-0, GS-10 and IS-10 are shown.

The former dependence was determined using Equation (8), while the specific electrical conductivity of grain boundaries was determined using two equations—Equation (12) for the GS-0 sinter and Equation (13) for all composite sinters. In the latter case, it was first necessary to calculate the grain boundary thickness (δ) of the investigated sinters from Equations (12)–(15) using impedance data and the mean grain size presented in Table 4. Table 7 lists the determined δ values together with the physical parameters used to describe equivalent circuit elements in the calculations, i.e., R_b_, A_b_, n_b_, R_gb_, A_gb_, n_gb_, C_b_ and C_gb_. The δ values determined for the studied samples ranged from 0.4 to 2.72 nm. Aside from those for samples IS-1 and IS-15, these values were different than the values reported for tetragonal YSZ materials—4–6 nm (Ikuhara et al. [64]), 5.0 nm (Guo and Zhang [65], Lee and Kim [66]). As can be seen in Figure 8, the electrical conductivity of the grain interiors (σ_b_) was significantly higher than the specific electrical conductivity of grain boundaries (σ_sp,gb_) across the entire range of temperatures used for the measurements. 

For the GS-0 sample, σ_b_ at 973 K was higher than σ_sp,gb_ by 1 order of magnitude, and at 573 K, it was higher by 2.5 orders of magnitude. This shows that σ_sp,gb_ increased at a higher rate than σ_b_. Although the significantly lower grain boundary conductivity suggests that it should be the most significant factor inhibiting oxygen transport, the latter might also be limited if diffusion through the grain boundaries is on a much smaller scale than via the bulk. 

As far as the differences in the σ_b_ plots for the three sinters selected for Figure 8 are concerned, GS-0 exhibited the highest value of this parameter across the entire temperature range, while IS-10 was characterized by the lowest one; the differences were nevertheless not very pronounced. With regard to σ_sp,gb_ of GS-0, the situation was similar; of the sinters modified with alumina (GS-10 and IS-10), the GS-10 sinter exhibited lower electrical conductivity than IS-10 in the temperature range of 673–973 K, whereas below 673 K the opposite was true.

**Figure 9:**
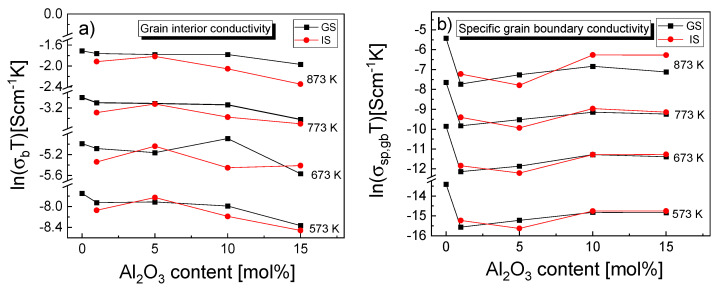
Dependence of the (**a**) grain interior electrical conductivity and (**b**) specific grain boundary electrical conductivity on alumina content in samples, as determined for 573, 673, 773 and 873 K.

Figure 9 illustrates the effect that the concentration of alumina inclusions in the 3Y-TZP matrix had on both σ_b_ and σ_sp,gb_ in the case of both series of sinters and for several measurement temperatures.

The grain interior electrical conductivity (σ_b_) for samples GS-1 to GS-10 remained at a nearly constant level and then dropped sharply as the alumina content reached 15 mol% (GS-15). The only exception was an increase in the σ_b_ component of conductivity for the GS-10 sample, observed at 673 K. For the IS sinters, σ_b_ increased when going from 1 mol% of Al_2_O_3_ to 5 mol% and then dropped as alumina content increased further, with a very slight exception again observed for the sample with 10 mol% and the temperature of 673 K (Figure 9a). 

Of the investigated composite samples (i.e., samples other than GS-0), those with the alumina content of 10 and 15 mol% exhibited the highest value of the specific electrical conductivity of grain boundaries (σ_sp,gb_) at all considered temperatures. Furthermore, the IS samples generally exhibited higher σ_sp,gb_ than the GS samples, especially at higher measurement temperatures. 

Overall, the segregation of aluminum at grain boundaries in the matrix of the 3Y-TZP samples causes their conducting properties to worsen. This effect is not offset by the previously mentioned scavenging of silica impurities by alumina. The aluminum ions might increase the height of the Schottky barrier and thereby increase the activation energy of conductivity in the space charge region [29]. Activation energy is a property that may be used to evaluate thermally activated phenomena—including those connected with ionic conductivity in zirconia-based electrolyte materials. The activation energy of total electrical conductivity (E_a(tot)_) as well as the values of its grain interior (E_a(b)_) and grain boundary (E_a(sp_._gb)_) components were determined for all studied symbols using Equation (10); the results are presented in Table S2 (Supplementary Materials). The error in these calculations did not exceed 0.01 eV. The activation energy of total conductivity (E_a(tot)_) was in the range of 0.79–0.82 eV for the GS sinters and in the range of 0.76–0.82 eV for the IS ones. Similar values have been reported in the literature for this type of ceramics [30,31,40,62,63]. GS-15 had the highest E_a(tot)_ value of the samples from the first group, while in the second group the highest value was found for IS-10. Alumina acts as an insulator, which is why higher concentrations of this compound affect activation energy in this way. The activation energy of the grain boundary conductivity was higher than the activation energy of the grain interior conductivity, which is consistent with the results presented in [31,63,67]. In the case of GS sinters, the activation energy values of grain interior and grain boundary conductivity were comparable regardless of the concentration of Al_2_O_3_ inclusions. Greater differences were observed for the IS series. The highest activation energies of each of the two components of electrical conductivity were found for IS-10.

**Figure 10:**
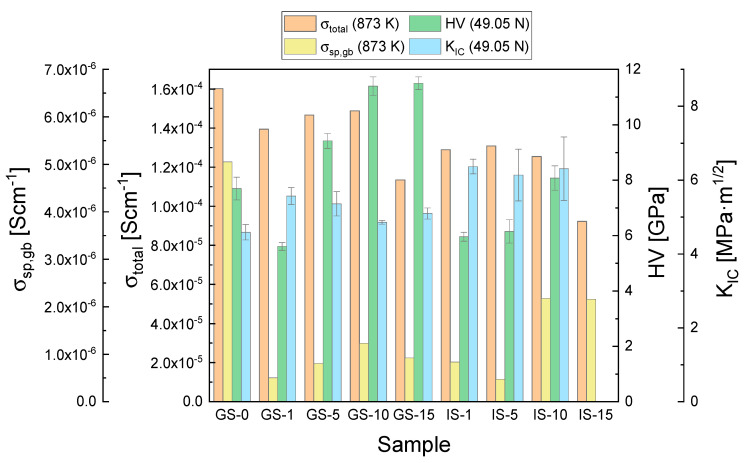
Most significant properties of the studied GS and IS sinters: the total electrical conductivity and specific grain boundary conductivity measured at 873 K, Vickers hardness values (HV), and the critical stress intensity factors (K_IC_) at a load of 49.05 N.

The parameters that were determined for all of the investigated sinters as part of the present work and can be considered to be the most significant—the total electrical conductivity and specific grain boundary conductivity measured at 873 K, Vickers hardness values (HV) and the critical stress intensity factors (K_IC_) at a load of 49.05 N—are collected in Figure 10 to provide an overall comparison of the studied materials.

When all of the presented data are taken into account, the sample that may be considered to exhibit the most desirable electrical and mechanical properties is the composite with 10 mol% of Al_2_O_3_ introduced in the 3Y-TZP matrix via the gelatin method (GS-10). This material is the best candidate for application as an electrolyte material in solid oxide fuel cells designed for operation at temperatures no higher than 873 K (IT-SOFCs).

## 4. Conclusions

✓ Zirconia (3-YSZ) powder stabilized with 3 mol% of yttria with nano-sized crystallites containing both tetragonal and monoclinic phases was obtained via the gelatin method and then calcinated. This powder was shown to be suitable for the preparation of dense 3Y-TZP/Al_2_O_3_ composite sinters with different amounts of Al_2_O_3_ (1, 5, 10 and 15 mol%) added either during the preparation of the 3-YSZ powder or via impregnation using an alcohol solution of aluminum nitrate.
Two series of the 3Y-TZP/Al_2_O_3_ composite samples obtained after sintering the green bodies for 2 h in air at 1773 K in air varied in terms of phase composition depending on the amount of added alumina and the applied addition procedure. Tetragonal zirconia was the predominant phase in all studied samples, which also contained minor amounts of the monoclinic phase. The lattice constants of the tetragonal phase in the 3Y-TZP samples did not depend on the alumina content.
✓ Morphological observations showed that the matrix composed of 3Y-TZP had a fine-crystalline character and consisted of average grains with a size in the range of ca. 0.38–0.42 µm. In the 3Y-TZP/Al_2_O_3_ composite samples with up to 5 mol% of alumina, uniformly distributed inclusions were found, while in the samples that had higher alumina content, isolated alumina clusters with a non-uniform distribution formed. Increased alumina content was generally associated with a slight decrease in grain size, which indicates that adding Al_2_O_3_ to the 3Y-TZP material inhibited grain growth. The opposite tendency was observed in the case of the mean alumina inclusion size. The obtained sinters varied considerably in terms of relative density (76–99%). The sinter with 10 mol% of alumina was the least porous one.
✓ Increased concentration of alumina in the 3Y-TZP matrix was generally associated with increased hardness of the composite material. The sintered samples that had alumina introduced during powder synthesis were found to exhibit higher hardness than the samples modified via impregnation. The brittle fracture toughness at a load of 49.05 N was in the range of 4.6–6.4 MPa·m^1/2^. The presence of alumina inclusions increased the value of the critical stress intensity factor (K_IC_). The fracture resistance of the samples modified via impregnation was higher by ca. 30%.
✓ The electrical conductivity was measured via EIS, and the total electrical conductivity as well as grain interior and specific grain boundary conductivity were all found to increase with temperature. Conversely, all of these parameters decreased for higher concentrations of added alumina. Conductivity was also affected by the method applied to introduce alumina into the matrix. The grain interior conductivity was significantly higher than the specific electrical conductivity of the grain boundaries. The sintered sample with no alumina addition exhibited the highest electrical conductivity across the entire range of measurement temperatures.

Conductivity was slightly lower in samples with up to 10 mol% of Al_2_O_3_ but was the lowest for the two sintered samples with the highest alumina content. For the samples with 1–10 mol% of Al_2_O_3_ added during powder synthesis, the differences in grain interior conductivity were negligible, while the tendency for the samples modified via impregnation was slightly more complex. As far as the specific electrical conductivity of grain boundaries is concerned, the composite sinters prepared from samples with alumina added during powder synthesis generally exhibited lower values than the second group of composite samples, with the exception of the sample with a 5 mol% addition. However, the presence of alumina inclusions had a negative impact on the grain boundary conductivity for both series of samples.

## Figures and Tables

**Table 1:** Sample designation (x—alumina content [mol%]).

Sample Type	Sample Preparation Procedure	
	Gelatin Method	Impregnation Method
Powder	GP-x	IP-x
Sinter	GS-x	IS-x

**Table 2:** Mass fractions of the tetragonal (t-ZrO_2_) and monoclinic (m-ZrO_2_) zirconia phases and the size of the tetragonal phase crystallites for two series of studied powders obtained via 2 h of calcination in air at 873 K.

Sample	Mass Fraction [%]		Crystallite Size (D_(011)_) [nm]
	t-ZrO_2_	m-ZrO_2_	
GP-0	88	12	13.1
GP-1	52.1	47.9	6.9
GP-5	69.2	30.8	5.8
GP-10	76.9	23.1	6.7
GP-15	68.2	31.8	8.0
IP-1	80.1	19.9	11.6
IP-5	76.6	23.4	11.1
IP-10	75.4	24.6	8.7
IP-15	72.8	27.2	8.6

**Table 3:** Mass fractions of the phases identified for samples sintered for 2 h in air at 1773 K as well as the lattice constants of the t_1_ and t_2_ tetragonal phases and the corresponding degree of tetragonality.

Sample	Mass Fraction of the Detected Phases [%]				Lattice Constants of Tetragonal Phases [nm]		Tetragonality (c/a)	
	t_1_-ZrO_2_	t_2_-ZrO_2_	m-ZrO_2_	Al_2_O_3_	t_1_	t_2_	t_1_	t_2_
GS-0	89.4	10.6	−	−	a = 0.360500 c = 0.517881	a = 0.36239 c = 0.51543	1.4366	1.4223
GS-1	80.80	17.50	0.64	1.10	a = 0.360466 c = 0.517853	a = 0.362408 c = 0.51526	1.4366	1.4218
GS-5	77.80	16.50	0.71	5.00	a = 0.360513 c = 0.517970	a = 0.362451 c = 0.51540	1.4368	1.4220
GS-10	75.90	15.10	0.42	8.50	a = 0.30468 c = 0.517944	a = 0.362406 c = 0.51528	1.4369	1.4218
GS-15	70.20	15.10	0.51	14.20	a = 0.360517 c = 0.518023	a = 0.36248 c = 0.51544	1.4369	1.4220
IS-1	86.40	12.40	0.44	0.80	a = 0.360453 c = 0.517850	a = 0.36238 c = 0.51527	1.4367	1.4219
IS-5	82.80	11.20	0.54	5.50	a = 0.36046 c = 0.517908	a = 0.36241 c = 0.51534	1.4368	1.4220
IS-10	76.00	10.40	0.37	13.20	a = 0.360511 c = 0.518047	a = 0.36249 c = 0.51743	1.4370	1.4219
IS-15	69.20	9	4.58	17.20	a = 0.360524 c = 0.518047	a = 0.36251 c = 0.51573	1.4369	1.4227

**Table 4:** The mean size of the grains of the 3Y-TZP matrix and the alumina inclusions as well as their circularity for unmodified 3Y-TZP (GS-0) and two series of 3Y-TZP/Al_2_O_3_ composites with alumina added using two different methods (gelatin method—GS samples; and impregnation—IS samples).

Sample	Mean Grain Size of 3Y-TZP Matrix [µm]	Circularity of 3Y-TZP Matrix Grains	Mean Size of Alumina Inclusions [µm]	Circularity of Alumina Inclusion
GS-0	0.380 ± 0.146	0.66 ± 0.10	−	−
GS-1	0.377 ± 0.171	0.66 ± 0.12	0.35 ± 0.09	0.57 ± 0.16
GS-5	0.410 ± 0.145	0.66 ± 0.10	0.46 ± 0.19	0.60 ± 0.15
GS-10	0.381 ± 0.147	0.66 ± 0.11	0.59 ± 0.18	0.68 ± 0.13
GS-15	0.375 ± 0.111	0.68 ± 0.11	0.58 ± 0.23	0.61 ± 0.17
IS-1	0.419 ± 0.157	0.71 ± 0.09	0.41 ± 0.17	0.53 ± 0.17
IS-5	0.417 ± 0.152	0.73 ± 0.11	0.42 ± 0.10	0.72 ± 0.14
IS-10	0.414 ± 0.144	0.71 ± 0.10	0.54 ± 0.24	0.60 ± 0.17
IS-15	0.410 ± 0.140	0.71 ± 0.09	0.62 ± 0.20	0.63 ± 0.17

**Table 5:** The apparent, theoretical and relative densities as well as the corresponding total porosity values for the studied samples obtained after 2 h of sintering in air at 1773 K.

Sample	Apparent Density [g∙cm^−3^]	Theoretical Density [g∙cm^−3^]	Relative Density [%]	Total Porosity [%]
GS-0	5.1891	6.0752	85.42	14.58
GS-1	4.5735	6.0554	75.52	24.48
GS-5	5.2143	5.9511	87.62	12.38
GS-10	5.7446	5.8238	98.64	1.36
GS-15	5.2343	5.6998	91.83	8.17
IS-1	4.8192	6.0554	79.59	20.41
IS-5	4.7083	5.9511	79.12	20.88
IS-10	5.0801	5.8238	87.23	12.77
IS-15	4.6157	5.6998	80.98	19.02

**Table 6:** Vickers hardness values (HV) for loads of 9.81, 29.42 and 49.05 N as well as the critical stress intensity factors (K_IC_) for loads of 29.42 and 49.05 N, as measured for the investigated sinters obtained via 2 h of thermal treatment in air at 1773 K.

Sample	HV [GPa]			K_IC_ [MPa∙m^1/2^]	
	9.81 [N]	29.42 [N]	49.05 [N]	29.42 [N]	49.05 [N]
GS-0	7.11 ± 0.27	7.61 ± 0.41	7.70 ± 0.04	4.32 ± 0.21	4.59 ± 0.21
GS-1	5.45 ± 0.06	5.50 ± 0.14	5.61 ± 0.07	−	5.57 ± 0.23
GS-5	9.50 ± 0.40	9.62 ± 0.27	9.42 ± 0.21	5.30 ± 0.74	5.36 ± 0.33
GS-10	10.86 ± 0.26	11.52 ± 0.33	11.44 ± 0.07	4.77 ± 0.24	4.86 ± 0.05
GS-15	10.86 ± 0.29	11.37 ± 0.23	11.50 ± 0.21	5.08 ± 0.28	5.10 ± 0.15
IS-1	5.95 ± 0.20	5.85 ± 0.16	5.96 ± 0.09	−	6.37 ± 0.20
IS-5	6.05 ± 0.75	5.78 ± 0.42	6.15 ± 0.38	−	6.14 ± 0.70
IS-10	8.32 ± 0.50	8.10 ± 0.44	8.08 ± 0.46	−	6.31 ± 0.86

**Table 7:** Grain boundary thickness (δ) in the investigated sinters, as calculated using Equations (12)–(15) as well as a number of physical parameter (R_b_, A_b_, n_b_, R_gb_, A_gb_, n_gb_, C_b_ and C_gb_) corresponding to equivalent circuit elements.

Sample	R_b_ [Ω]	A_b_	n_b_	R_gb_ [Ω]	A_gb_	n_gb_	C_b_ [F]	C_gb_ [F]	δ [nm]
GS-0	52155	7.96E-11	0.88734	106520	8.99E-09	0.83642	1.6508E-11	2.3072E-09	2.72
GS-1	64557	5.24E-11	0.90518	140920	1.51E-08	0.78778	1.401E-11	2.8772E-09	0.40
GS-5	53626	7.38E-11	0.8936	105590	9.49E-09	0.84623	1.6769E-11	2.7041E-09	0.54
GS-10	77189	4.94E-11	0.90943	134960	6.11E-09	0.84229	1.4265E-11	1.6158E-09	0.72
GS-15	116380	3.27E-11	0.92375	175800	3.84E-09	0.85034	1.1662E-11	1.0611E-09	0.94
IS-1	70557	4.32E-11	0.91683	138140	1.29E-08	0.76857	1.3646E-11	1.909E-09	0.64
IS-5	60504	6.45E-11	0.89095	158560	1.46E-08	0.78633	1.4044E-11	2.8064E-09	0.45
IS-10	93453	4.15E-11	0.91572	166990	5.12E-09	0.82301	1.3183E-11	1.1209E-09	1.03
IS-15	114530	4.23E-11	0.91331	227080	4.15E-09	0.80581	1.3253E-11	7.747E-10	1.50

## Data Availability

The data presented in this study are available on request from the corresponding authors.

## Supplementary Materials

The following supporting information can be downloaded at: https://www.mdpi.com/article/10.3390/ma15062101/s1, Figure S1: TG/DTA curves recorded during the thermal treatment of the 3-YSZ gel precursor with no alumina addition. Figure S2: Dilatometric curves for two series of powders after 1 h of calcination in air at 873 K. Figure S3: Tetragonality of the t_1_ and t_2_ tetragonal phases as a function of alumina content for the two investigated series of sinters. Figure 4S: Mean size in samples sintered for 2 h in air at 1773 K as a function of alumina content: (a) grains of the 3Y-TZP matrix and (b) Al_2_O_3_ inclusions; Table S1: Temperature ranges associated with expansion and shrinkage, as determined for two series of powders after 1 h of calcination in air at 873 K. Table S2: Activation energy of conduction with respect to total conductivity (E_a(tot)_) as well as grain interior (E_a(b)_) and specific grain boundary conductivity (E_a(sp.gb)_), as calculated for the studied 3Y-TZP and 3Y-TZP/Al_2_O_3_ samples obtained after 2 h of sintering in air at 1773 K. Refs [68,69,70,71,72] are cited in the SM.
