# Supplementary material for: Effects of Aluminum Oxide Addition on Electrical and Mechanical Properties of 3 mol% Yttria-Stabilized Tetragonal Zirconia Electrolyte for IT-SOFCs
Source: Materials (Basel). 2022 Mar 12;15(6):2101. doi: 10.3390/ma15062101 (PMC8956071; doi:10.3390/ma15062101)
Supplement: Supplementary file 1 [file materials-15-02101-s001.zip › materials-1518415-supplementary.pdf]

Effects of aluminum oxide addition on electrical and mechanical properties of 3 mol% yttria-stabilized tetragonal zirconia electrolyte for IT-SOFCs

Justyna Chłędowska (Pleśniak), Jan Wyrwa, Mieczysław Rękas, Tomasz Brylewski*

AGH University of Science and Technology, Faculty of Materials Science and Ceramics, al. Mickiewicza 30, 30-059 Kraków, Poland

1. Characterization of the samples

The density of alumina assumed for the calculations was d_{Al2O3} =3.987 g·cm^{-3}. For sinters consisting of 3Y-TZP (GS-0), the theoretical density of $d_{3Y\text{-}TZP}$ =6.0752 g·cm^{-3} was applied, which had been calculated as follows:

$$d_{XRD} = \frac{2m_{3Y-TZP}}{V_{unit\ cell}} \tag{1S}$$

where: $m_{3Y\text{-}TZP}$ – mass of 3Y-TZP molecule [g], $V_{unit\ cell}$ – unit cell volume [cm^3]. The number 2 corresponds to the number of tetragonal zirconia molecules in the unit cell. The mass of the 3Y-TZP molecule can be calculated from the following equation:

$$m_{3Y-TZP} = \frac{M_{3Y-TZP}}{6.02214 \cdot 10^{23}} \tag{2S}$$

where: $6.02214 \cdot 10^{23}$ – Avogadro's number, $M_{3Y\text{-}TZP}$ – molar mass of 3Y-TZP.

According to the assumed mechanism underlying the formation of a solid solution consisting of ZrO_2 with x mol% of Y_2O_3:

$$Y_2O_3 \rightarrow 2Y'_{Zr} + 3O_O^x + V_O^{\cdot\cdot} \tag{3S}$$

the chemical formula of a 3YSZ molecule is:

$$Zr_{0.94}Y_{0.06}O_{2-0.03}(V_O)_{0.03} \tag{4S}$$

and thus using the appropriate atomic mass values for calculations we get:

$$M_{3Y-TZP} = 0.94 \cdot 91.224 + 0.06 \cdot 88.906 + 1.97 \cdot 15.999 = 122.60355 \tag{5S}$$

When this value is used with equation (2S), the result is as follows:

*Corresponding author at: AGH University of Science and Technology, Faculty of Materials Science and Ceramics, Al. Mickiewicza 30, 30-059 Krakow, Poland, Tel.: +48 12 617 5229; fax.: +48 12 6172493. E-mail address: brylew@agh.edu.pl (T. Brylewski)

$$m_{3Y-TZP} = \frac{122.60355}{6.02214 \cdot 10^{23}} = 20.3588 \cdot 10^{-23}\ g \quad (6S)$$

The volume of a tetrahedral unit cell ($V_{unit\ cell}$) can be calculated based on the XRD data obtained for the studied sinters, and it is:

$$V_{unit\ cell} = a^2 \cdot c = 6.73 \cdot 10^{-23}\ cm^3 \quad (7S)$$

Substituting the variables in equation (5) with the values obtained from equations (6S) and (7S) yields $d_{XRD} = 6.0752\ g \cdot cm^{-3}$.

2. Physicochemical properties of the precursor gel and powders

The results of DTA/TG thermal analyses for the 3-YSZ gel precursor with no alumina addition suggest that in this case there were several stages of decomposition. As shown in Fig. 1S, an exothermic effect with an associated sample mass loss is observed during each stage.

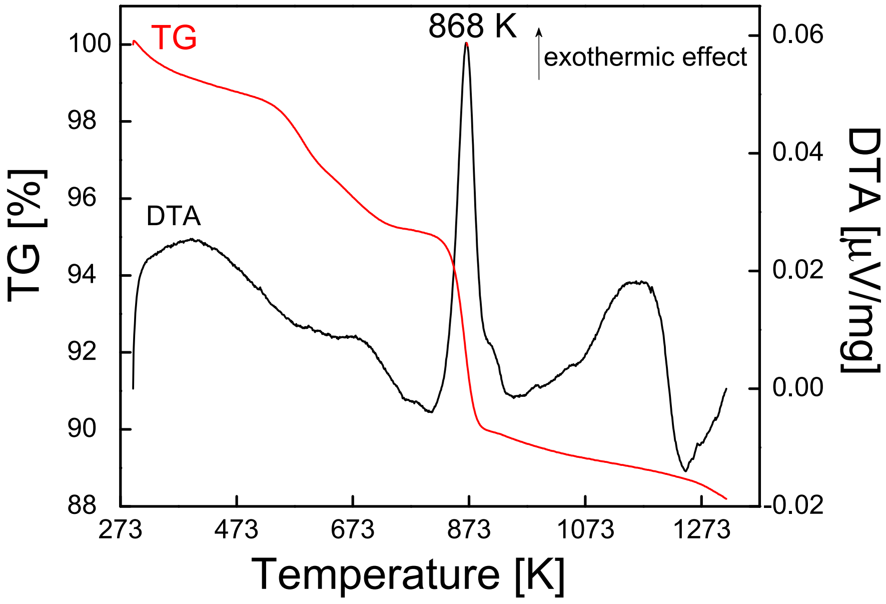

Figure S1. TG/DTA curves recorded during the thermal treatment of the 3-YSZ gel precursor with no alumina addition.

In the first stage, which corresponded to the temperature range of ca. 333-448 K, the gel was dehydrated and nitrate groups underwent decomposition, which resulted in a mass loss of 1.1%. As indicated by literature data [1-5], amorphous ZrO_2 and Y_2O_3 phases may precipitate in the range of 550-700 K. This stage also involves the decomposition of aminoacid fragments that easily degrade, mostly prolines; the associated mass loss was 3.82%. Yet another stage, which manifested itself as a pronounced exothermic peak, was related to the complete

degradation of the remaining aminoacids, including glycine [3]. It was during this stage that zirconia crystallized [1-4]. The associated mass loss was the highest observed, i.e. 5.5%. The last stage was observed for the temperature range of 1073-1273 K, and involved the growth of tetragonal zirconia crystals, as suggested by literature data [5]. During the performed thermal treatment, the precursor gel lost 11.86% of its initial mass in total. Based on the thermal analyses conducted for the gel, it was established that the lowest applied calcination temperature would be 873 K.

To determine the conditions in which the green bodies would be sintered, a dilatometric analysis was performed; the results, namely the shrinkage kinetics, are listed in Fig. 2S.

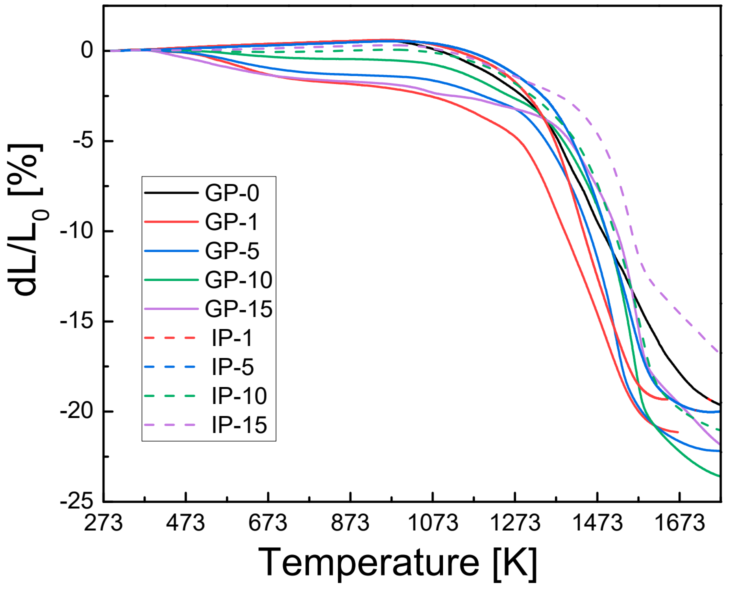

Figure S2. Dilatometric curves for two series of powders after 1 h of calcination in air at 873 K.

The $\Delta L/L_o=f(T)$ dependence (where ΔL – the change in the linear dimensions, L_o – the length of the sample at the starting temperature) was used to establish the range of temperatures in which the materials undergo expansion and shrinkage and to calculate the shrinkage values for the applied green bodies. The corresponding data are shown in Table 1S.

The GP-0 sample and the samples from the IP series undergo thermal expansion in the first heating stage; the observed expansion is ca. 0.5% for the temperature range of 293-960 K. The GP samples modified via an Al_2O_3 addition also expand very slightly, i.e. by about 0.05%; this expansion takes place up to a temperature of ca. 370 K. When the temperatures exceed the upper value of either of the afore-mentioned ranges, the sintering of the green

starts, which is associated with systematic shrinkage all the way to 1773 K – the highest temperature reached during the measurements (Table 1S).

Table S1. Temperature ranges associated with expansion and shrinkage, as determined for two series of powders after 1 h of calcination in air at 873 K.

Sample	Temperature range for expansion [K]	Temperature range for shrinkage [K]	Shrinkage [%]
GP-0	293-939	939-1772	19.6
GP-1	293-356	356-1685	21.2
GP-5	293-365	365-1772	22.2
GP-10	293-390	390-1773	23.6
GP-15	293-360	360-1773	21.8
IP-1	293-964	964-1643	19.3
IP-5	293-977	977-1748	20.0
IP-10	293-965	965-1773	21.0
IP-15	293-969	969-1773	16.8

The dilatometric curves presented in Fig. 2S indicate that shrinkage depends to a very large degree on the type of investigated material. The green bodies obtained using the GP powders were sintered over a wider range of temperatures than in the case of the IP series and the GP-0 sample. This can be attributed to the powders' crystallite size. Also worth noting is the fact that the green bodies from the GP series powders were characterized by a higher degree of shrinkage. The lowest level of shrinkage, i.e. ca. 17%, was observed for the IP-15 green body, which suggests that the highest applied temperature (1773 K) was insufficient to obtain a fully compacted sinter. The highest level of shrinkage (ca. 24%) was observed for the GP-10 sample. The analysis of the dilatometric curves proved that the selected sintering temperature made it possible to obtain sinters with the best possible density.

3. Physical characteristics of the obtained sinters

3.1. Structure

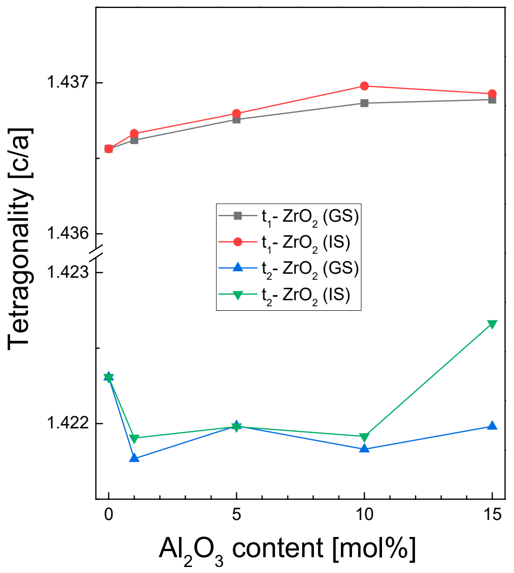

Figure S3. Tetragonality of the t_1 and t_2 tetragonal phases as a function of alumina content for the two investigated series of sinters.

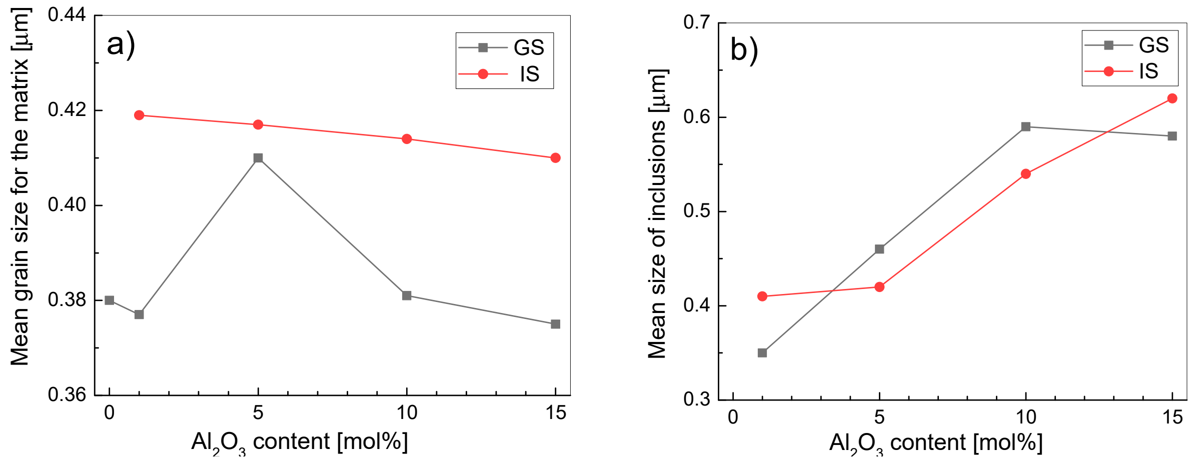

Figure S4. Mean size in samples sintered for 2 h in air at 1773 K as a function of alumina content: a) grains of the 3Y-TZP matrix and b) Al_2O_3 inclusions.

3.2. Electrical properties

Table S2. Activation energy of conduction with respect to total conductivity ($E_{a(tot)}$) as well as grain interior ($E_{a(b)}$) and specific grain boundary conductivity ($E_{a(sp.gb)}$), as calculated for the studied 3Y-TZP and 3Y-TZP/Al_2O_3 samples obtained after 2 h of sintering in air at 1773 K.

**Sample**	**Activation energy [eV]**		
	$E_{a(tot)}$	$E_{a(b)}$	$E_{a(sp.gb)}$
GS-0	0.80±0.02	0.79±0.02	1.14±0.07
GS-1	0.79±0.03	0.79±0.03	1.15±0.06
GS-5	0.80±0.02	0.79±0.02	1.16±0.07
GS-10	0.79±0.03	0.79±0.03	1.15±0.05
GS-15	0.82±0.02	0.82±0.02	1.15±0.05
IS-1	0.80±0.02	0.79±0.02	1.19±0.06
IS-5	0.76±0.03	0.76±0.03	1.16±0.06
IS-10	0.82±0.02	0.80±0.02	1.26±0.08
IS-15	0.79±0.03	0.78±0.03	1.14±0.04

References

1. Samdi, A.; Durand, B.; Roubin, M.; Daoudi, A.; Taha, M.; Paletto, J.; Fantozzi, G. Pressing and sintering behaviour of yttria stabilized zirconia powders prepared from acetate solutions. J. Eur. Ceram. Soc. 1993, 12, 353-360.
2. Govindarajan, S.; Dusane, R.O.; Joshi, S.V. In situ particle generation and splat formation during solution precursor plasma spraying of yttria-stabilized zirconia coatings. J. Am. Ceram. Soc. 2011, 94, 4191-4199.
3. Oliveira, F.S.; Pimentel, P.M.; Oliveira R.M.P.B.; Melo. D.M.A.; Melo, M.A.F. Effect of lanthanum replacement by strontium in lanthanum nickelate crystals. Mater. Lett. 2010, 64, 2700-2703.
4. Figueredo, G.; Melo A.F.C.; Medeiros, R.; Silva F.M.; Pimenta, H.M.; Melo, M.A.F.; Melo, D.M.A. Synthesis of MgAl2O4 by gelatin method: Effect of temperature and time of calcination in crystalline structure. Mat. Res. 2017, 20, 254-259.
5. Carpio, P.; Candidato, R.T.; Pawlowski, L.; Dolores Salvador, M. Solution concentration effect on mechanical injection and deposition of YSZ coatings using the solution precursor plasma spraying. Surf. Coat. 2019, 371, 124-130.
